# Perinatal and infant mental health care in Austria: Mapping of existing prevention, screening, and care services

**DOI:** 10.1007/s40211-024-00516-0

**Published:** 2024-12-30

**Authors:** I. Zechmeister-Koss, C. Hörtnagl, Astrid Lampe, J. L. Paul

**Affiliations:** 1 Austrian Institute for Health Technology Assessment GmbH, Garnisongasse 7/20, 1090 Vienna, Austria; 2 Department for Psychiatry, Psychotherapy, Psychosomatics and Medical Psychology, Medical University Innsbruck, Anichstraße 35, 6020 Innsbruck, Austria; 3 Ludwig Boltzmann Institute for Rehabilitation Research, Reizenpfenninggasse 1, 1140 Vienna, Austria; 4 VAMED Rehabilitation Montafon, Wagenweg 4a, 6780 Schruns, Austria

**Keywords:** Perinatal mental illness, Screening for perinatal mental illness, Prevention, Early identification, Perinatal psychiatric care, Peripartale psychische Erkrankung, Versorgung peripartaler psychischer Erkrankungen, Prävention, Früherkennung, Psychiatrische Betreuung in der Peripartalperiode

## Abstract

**Purpose:**

Perinatal mental illness (PMI) is one of the major health problems during pregnancy and one year after birth (the perinatal period), with robust evidence of its potentially detrimental effects on the parent’s and child’s health. Many countries have prioritised perinatal and infant mental health care (PIMHC). In Austria, it is currently unknown how many services are available in which region. The paper aims to map the current PIMHC landscape.

**Methods:**

Using publicly accessible sources, such as health reports or organisation websites and supplementary information from experts, we collected data on eight characteristics of services to prevent, early identify, treat or support parents with a PMI. We extracted the information into tables, narratively summarised the results and presented a geographical visualisation of service availability.

**Results:**

While there is currently no standardised nationwide systematic screening for PMI in place, there are a variety of services to support and treat parents with a PMI of different severity in Austria. However, there are large regional variations and gaps in care, particularly regarding specialised PIMHC and trained staff, leading to unequal access. PIMHC primarily addresses mothers and involves many, mostly public, providers and funding sources.

**Conclusion:**

There is an urgent need to reduce the regional disparities regarding specialised PIMHC, ensuring adequate referrals and treatment and reducing inequalities in access to care. The results also call for a national strategy and defined political, administrative and service provider responsibilities based on international evidence-based recommendations. Investing in the training of staff and defined care pathways seems warranted.

## Abstract (de)

**Hintergrund:**

Peripartale psychische Erkrankungen (PMI) gehören zu den häufigsten Gesundheitsproblemen während der Schwangerschaft und im ersten Jahr nach der Geburt (Peripartalperiode). Ihre potenziell negativen Auswirkungen auf die Gesundheit von Eltern und Kind sind eindeutig belegt. Viele Länder haben dem Thema „Perinatal and Infant Mental Health Care“ (PIMHC) Priorität eingeräumt. In Österreich ist derzeit nicht bekannt, welche Angebote in welcher Region verfügbar sind. Die Arbeit präsentiert eine Bestandsaufnahme der derzeitigen Angebotslandschaft.

**Methode:**

Anhand von öffentlich zugänglichen Quellen, wie Websites von Organisationen, und ergänzenden Informationen von Expert:innen haben wir Daten zu 8 Merkmalen von Angeboten zur Prävention, Früherkennung, Behandlung oder Unterstützung von Eltern mit einer PMI erhoben. Die in Tabellen extrahierten Detailinformationen wurden narrativ zusammengefasst. Es erfolgte eine geographische Visualisierung der verfügbaren Angebote.

**Ergebnis:**

Während es derzeit kein landesweit einheitliches systematisches Screening auf PMI gibt, existieren quer durch Österreich eine Vielzahl von Angeboten zur Unterstützung und Behandlung von Eltern mit PMI unterschiedlichen Schweregrades. Allerdings bestehen große regionale Unterschiede und Lücken in der Versorgung, insbesondere in Bezug auf spezialisierte PIMHC und geschultes Personal, was zu ungleichem Zugang führt. PIMHC richtet sich in erster Linie an Mütter und umfasst viele, meist öffentliche Anbieter und Finanzierungsquellen.

**Schlussfolgerung:**

Es besteht Bedarf, die regionalen Unterschiede bei der spezialisierten PIMHC zu verringern, angemessene Koordination und Behandlungen zu gewährleisten und Ungleichheiten beim Leistungszugang abzubauen. Hilfreich dafür scheinen eine nationale Strategie und die Definition von Zuständigkeiten in Politik, in Verwaltung und bei Leistungserbringern auf der Grundlage internationaler evidenzbasierter Empfehlungen sowie Investitionen in Ausbildung des Personals und in definierte Versorgungspfade.

## Background

Perinatal mental illness (PMI) is a common and serious complication during pregnancy and the first year after birth. It affects around 1 in 5 mothers [1] and 1 in 10 fathers worldwide [2]. It can also impact both parents concurrently, with, for example, up to 3% of couples being jointly affected by perinatal depression [3]. The most common types of PMI are depression and anxiety disorders, with a prevalence of about 15% of women. Serious mental health problems requiring hospital admission are less common [4].

There is strong evidence that PMI contributes to maternal mortality and adverse neonatal outcomes. Immediate effects can include complications during pregnancy or birth, difficulties in the attachment between parent and baby, behavioural or emotional problems of the child, and an increased risk of suicide or other causes of death of the parent, or infant mortality. In the long term, children are at higher risk of mental and physical illness, and their healthy development may be significantly impaired. The risk for adverse child outcomes can persist into late adolescence [5–9]. Recent evidence also shows long-term detrimental health impacts for mothers [10]. Furthermore, there are serious economic consequences for society; the largest proportion of costs relates to adverse impacts on the child [11]. To prevent the adverse consequences of perinatal mental illness, it is therefore essential to identify PMI early and to provide rapid and effective care [4].

In several countries, the prevention, early identification and care for PMI have been prioritized and systematically expanded [12]. For Austria, there is neither a national strategy nor a national care model for perinatal and infant mental health. More specifically, we lack a general overview of currently available services. This paper will address this knowledge gap by mapping the current service landscape to provide a rational base for further planning and improving perinatal and infant mental health care (PIMHC) in Austria.

## Methods

### Terms, definitions and scope

We classified formal services alongside a prevention and care continuum, as presented in a literature review [12]. We used the following categories: (1) primary prevention (including raising awareness on the prevalence and risk factors, counselling, and addressing perinatal mental health literacy), (2) early detection/screening and (3) care and treatment of parents with perinatal mental health problems or manifest illness. Furthermore, we distinguished between specialist PIMHC services (defined by the infrastructure, focus of the program or qualification of staff) and those that are used by parents with perinatal mental health problems among other target groups but do not have a perinatal mental health (treatment) focus, e.g. child and youth welfare services. Accommodation services that parents with a PMI may use, such as those for women affected by domestic violence or other out-of-home placements are excluded.

Within specialist services, we first included services for which the index patient is the parent with a mental health problem, such as mother–baby units for hospital treatment. Secondly, we considered services where the index patient is the infant who shows symptoms that may result from parental mental health problems (e.g. infant psychosomatic services). In addition to formal services, we searched for existing informal services, such as self-help groups. We considered services for treating parents with manifest diagnoses according to the International Classification of Diseases under the category ‘F’ (ICD-10 F00 to F99), but also those that address mental health concerns more broadly or where symptoms are mild or subclinical. We are using PIMHC as a summary term for the entire service spectrum.

### Data sources and analysis

Our database for the mapping was publicly accessible sources, such as health reports, websites of organisations or national statistics, which we identified by hand search. Key sources of information were national and regional governmental websites, websites of hospital providers and reports on public services (e.g. social services reports, child and youth welfare reports, health reports). Additionally, we used search terms on specific services in a Google search (e.g. ‘Mutter-Kind-Bett, ‘Schreiambulanz’). In case of information gaps, supplementary information was sought from practitioners in the field of (perinatal) mental health care and women’s health whom we contacted by email or telephone. Experts were identified via snowball sampling, starting with providers of services in the field of parental mental illness (e.g. ‘Pro Mente Kärnten’, ‘Kolibri’ in Vorarlberg) and women’s health (e.g. Frauengesundheitszentrum Graz, Wiener Büro für Frauengesundheit), complemented with heads of individual services (e.g. psychiatrists running mother–baby beds) who were named by others or identified in a web search. The search was conducted between October 2022 and January 2023. We extracted the information for each Austrian state into tables, which we structured according to the predefined categories described above (tables can be accessed at https://eprints.aihta.at/1437/1/HTA-Projektbericht_Nr.151.pdf). For each service, we collected information on eight characteristics (name of service, name of provider, type of provider, program content, capacities, primary target group, professional groups involved, funding). We narratively summarised the table information and prepared a geographical visualisation of service availability.

## Results

### Overview of service landscape

**Fig. 1:**
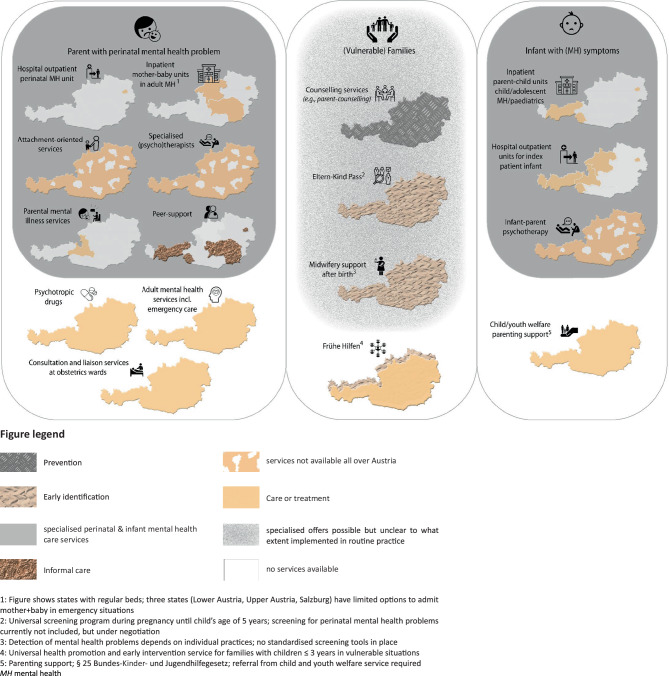
Perinatal and infant mental health care services landscape in Austria

Figure 1 demonstrates an overview of the service landscape. Overall, we identified a broad range of services across Austria with large regional variations. Alongside the prevention–early identification–care/treatment continuum, most services fall under the ‘care/treatment’ category, while we did not identify systematic prevention and universal early detection programs by January 2023. Although some services may be and—according to expert information—are increasingly used by fathers, we did not identify services that explicitly address perinatal mental health problems in fathers or partners, but services predominantly focus on mothers (e.g. by naming them ‘Mutter-Kind-Zentrum’). Except for Vienna, there was no explicit information on whether services were provided in languages other than German and websites describing the services were often restricted to German only.

### Prevention and early detection

If at all, per January 2023, prevention in the form of education about PMI only took place sporadically or at individual organisations, for example, if there is a personal interest in the topic on the part of the trainer at a course for expecting parents. However, there are many offers where such preventive work could be integrated, for example, as part of the ‘Familienberatungsstellen’ which are provided across Austria, the numerous ‘Eltern-Kind-Zentren’ or women’s counselling services (e.g. ‘Frauenzentrum Osttirol’). Indeed, in the revision of the Austrian national screening program during pregnancy and early childhood (‘Eltern-Kind-Pass’) such counselling is planned to be provided universally, but public information regarding details is missing to date [13].

Regarding screening, universal systematic screening processes covering all women during pregnancy and after birth to detect PMI are neither implemented in any of the nine Austrian states, nor at the national level. Such screening has long been recommended as part of the ‘Eltern-Kind-Pass’ [14]; however, negotiations on whether and how to integrate evidence-based screening tools are still ongoing.

On the regional level, the ‘Wiener Programm für Frauengesundheit’, funded by the Viennese government, has developed screening tools for professionals working in hospital outpatient departments of obstetrics and for gynaecologists and paediatricians working in private practice. In addition, they published a brief general guideline for professionals involved in care during pregnancy and after birth [15]. Similarly, the Styrian health fund has issued a short guidance document [16], however, without stating clear pathways or responsibilities. In general, using such guidance is voluntary, and it is unclear to what extent professionals apply them routinely in a standardised way. Other options for universal screening (after birth) are the midwifery services, whereby mothers are entitled to a defined number of home visits by a midwife for a defined period after birth. Currently, there are no standardised perinatal mental health screening measures in place for those visits, but midwives may address the topic individually. However, only five percent to one quarter of the midwives (depending on the region) have a contract with health insurance [17], meaning that their service is publicly funded.

In contrast to universal screening, a targeted screening is provided by the recently nationwide implemented service ‘Frühe Hilfen’, a low threshold health promotion and/or early intervention approach offered during pregnancy until the child is 3 years old, in case of need for support. Identifying mental health issues is part of their standardised assessment tools. The service also organises referrals to specialised treatment if a need is identified. Yet, families using the service represent only a small subgroup of all annual births.

### Care and treatment

#### When the parent is the index patient

Regarding care and treatment, we found some form of specialist PIMHC services in each state, yet with substantial disparities across and within states. Regarding hospital-based services (the highest level of care), we identified only one specialised hospital outpatient unit with regular opening hours in Vienna as of January 2023. In some cases (Wels), existing units had to be closed due to a lack of resources. In others (Tyrol, Styria) the foundation of units is planned or in the process of implementation. Sporadically, there are small-scale offers with restricted opening hours available (e.g. Klagenfurt hospital).

Furthermore, there are nine to ten formal inpatient mother–baby beds available in three Austrian states (Vienna: 4; Styria: 2; Upper Austria: 3 to 4). Four states have an option to admit mothers with their baby in an emergency (Lower Austria, Salzburg, Vorarlberg, Upper Austria), yet without permanent infrastructure and established perinatal mental health care teams. Three states (Tyrol, Carinthia, Burgenland) have no options to admit mothers with their babies. There are currently no specialised hospital daycare facilities or other forms of acute specialised treatment (e.g. home treatment) available. According to expert information, the number of mother–baby units is planned to be slightly increased in some states (e.g. in Salzburg/Schwarzenberg Klinikum).

Experts informed us that admission into all types of mother–baby units in adult mental health care facilities is usually subject to the mother still being able to care for the child. Professional care for infants is provided but organised differently, for example, by permanently employed or liaise infant nurses or by psychiatric nurses trained in infant care. Details on treatment approaches are not published on the hospitals’ websites. We, therefore, do not know to what extent special offers to address the mother–child interaction are available or whether mothers primarily receive routine adult mental health treatment and children are co-admitted, guaranteeing custody and childcare. If the mother’s mental health makes her unable to care for the infant, partners or other potential carers are asked to take custody. The infant may be admitted to the paediatric unit without such a carer. Mother–infant interaction programs are not possible in this situation.

On a lower level of care, we identified a variety of specialised psychotherapy and attachment-oriented services in the community setting. Examples of those are therapeutic offers for mothers with mental health problems in Tyrol and Vienna as part of the ‘Frühe Hilfen’ service, infant–parent psychotherapy services offered by specialised organisations (e.g. ‘Österreichische Gesellschaft für Kinder- und Jugendpsychotherapie’) or by therapists in private practices, and attachment-oriented therapies. The latter can be specific forms of therapy (e.g. body-oriented therapy) or counselling (emotion-oriented) or may also include parenting skill training and support in daily life. Examples of services are ‘Grow Together’ offered in Vienna or ‘Zoi’ provided in Tyrol. In one state (Salzburg), programs designed to support parents with a mental illness offer a scheme for the perinatal period (‘Verein Jojo’) or extend their target groups to include parents in the perinatal period (‘PrEKIDS’). As a low threshold service in case of mild symptoms, ‘Frühe Hilfen’ has been available across all states since 2023. This is also one of the few services which offer outreach care in the families’ homes.

In addition to specialist services, services are available that may be used by parents with a mental health problem in the perinatal period but that do not treat the health issue and/or do not have specially trained staff. These include general adult mental healthcare facilities and services, including pharmacological treatment and psychiatric/psychological consultation and liaison service (e.g. to support the obstetrics departments in case of a mother’s mental health problem during admission around birth). Some professionals working in those services may have special training or experience in treating PMI. However, this qualification is not publicly documented. Furthermore, several services in the social sector, most notably those provided by the child and youth welfare as part of the ‘Unterstützung der Erziehung’-scheme (e.g. ‘Sozialpädagogische Familienbetreuung’), are used to some extent by parents with a PMI. Often, their use is mandatory and subject to severe problems and referral from the child and youth welfare. Such services may ensure that the parent receives treatment elsewhere but focus on supporting parenting and avoiding child neglect.

#### When the infant is the index patient

Regarding specialised care where the infant is the index patient, we identified hospital outpatient units (e.g. for babies with excessive crying) and day-clinics for infant psychosomatic care (e.g. ‘Baby-Care-Ambulanz’ at the ‘Klinik Favoriten’ in Vienna) in five of the nine Austrian states (Salzburg, Tyrol, Upper Austria, Vienna, Vorarlberg). In some regions, infants with psychosomatic or mental health symptoms can be admitted to a paediatric or child and adolescent mental health care unit in hospitals with a parent (e.g. Hall in Tirol with six beds), but not if the parent has severe mental health symptoms.

### Informal support

We identified self-help groups with a focus on PMI in three Austrian regions (Styria, Tyrol, Vienna) on the websites of self-help associations: the drop-in ‘Selbsthilfegruppe für Mütter mit psychischen Belastungen nach der Geburt’ in Innsbruck, the Styrian group ‘postpartale Depression’ and the Viennese group ‘Mutterglück! Mutterglück?’ organised by ‘NANAYA (Zentrum für Schwangerschaft, Geburt und Leben)’. They meet once to twice per month, can be accessed free of charge, and two of them are supported by professionals.

### Service characteristics

#### Providers

The services are provided by a mix of public and private providers, whereby private providers outweigh the number of public providers. Public providers are mostly restricted to hospital-based services, whereas programs in other settings are generally offered by private providers. All private providers are nonprofit organisations.

The size of organisations in terms of service portfolio and geographical coverage varies, ranging from small organisations providing a service in a single district, to larger ones providing a greater variety of services and/or serving a larger geographical area. An example of the former is the organisation ‘ZOI’ in Tyrol, which primarily offers services to improve parent–infant attachment in one Tyrolean district (Kufstein). An organisation representing the latter is ‘Beratungsstellen ÖKIDS’, organised by the Austrian Society for Child and Adolescent Psychotherapy, which offers infant–parent psychotherapy in five states. Most organisations are based in one of the nine Austrian states.

#### Funding

With few exceptions (‘Eltern-Kind Pass’, ‘Frühe Hilfen’, ‘Familienberatung’), which are funded and governed at the national level, and some health insurance-funded outpatient services (midwifery support after birth), responsibility for the funding of most of the services identified rests with the regional governments and sometimes even with districts or city governments. Consequently, various funding sources and all types of public payers, funding health and social care services in Austria, are involved. It is noticeable that some programs offered within hospitals (usually outpatient services) are not funded via the regular hospital outpatient reimbursement scheme, but via separate funding arrangements, sometimes involving many sources, including project-based funding. Examples are the hospital outpatient units ‘FEM’ and ‘FEM Süd’ in Vienna, which are funded by at least five different sources (e.g. ‘Stadt Wien’, ‘Wiener Institut für Gesundheitsförderung’, ‘Österreichische Gesundheitskasse’).

In addition to public funding, private funding plays a role. Firstly, some services are in parts and, in rare cases, funded fully from donations from regional companies, charity organisations, or private donors (e.g. ‘Verein JoJo’ in Salzburg). Secondly, users must pay private fees or co-payments for some services to access them (e.g. prescription fee). However, in case of user charges, fees may depend on the economic situation of the user, and if required, services may be offered free of charge. Services for which up to full private payment can be required most often are psychotherapy services, except if they are provided under a contractual arrangement with a public funder (e.g. health insurance) or by an organisation which is publicly funded and provides psychotherapy by employed therapists.

Reimbursement schemes of providers, which are publicly funded, differ considerably across services and regions. Hospital inpatient services are funded via a diagnosis-related group (DRG) system, where interventions requiring specific resources are usually linked to a defined tariff covering costs. Yet, there is no specific reimbursement code for mother–child admissions in adult mental health care units. Hospital owners with mother–baby beds deal differently with this situation. Some have arranged to reimburse admission using a code from child and adolescent mental health admissions (‘Einheiten mit der Behandlungsform E/Eltern-Kind’). However, this code was abolished in 2021. Others use the standard DRGs from adult mental health to reimburse the costs for treating the mother, and the baby is coded as an ‘accompanying person’.

Outpatient services in private practices in the health care sector (e.g. outpatient psychiatrists, psychotherapists, midwives) are reimbursed via a tariff per service negotiated between the professional groups and the health insurance. In some cases, reimbursement is a mix of flat rates and tariffs per service. Reimbursement for organisations in the social sector which receive public funding varies. Some organisations have short- or long-term contractual arrangements (e.g. those funded by child and youth welfare), while others may receive subsidies or funding on a project basis. These arrangements are subject to the payer’s budgetary situation and the decision maker’s discretion. They are, therefore, linked to less financial security for providers than services funded via hospital funding schemes or health insurance tariffs.

#### Professional groups involved

Multiprofessional teams characterise the workforce in most of the services identified. Providers mentioned more than 40 professional groups or auxiliary staff on their websites. These include medical specialities (e.g. psychiatrists, gynaecologists), allied health professionals (e.g. nurses, midwives, psychologists, different types of therapists), pedagogues and educational specialists (e.g. social pedagogues), social workers, counsellors with diverse backgrounds and several allied professionals and auxiliary staff (e.g. life coaches, interpreters). Professional groups stated most often were psychologists and psychotherapists.

### Coordination and interdisciplinary exchange

According to the Austrian depression report [14], formal exchange between services and professional groups currently exists in Vienna. The network ‘Psychosoziale Gesundheit in der Schwangerschaft’ meets two to three times per year, aiming to improve PIMHC (including identifying gaps) and foster interdisciplinary training and exchange. Vienna has also established semi-formal referral pathways for professionals identifying a parent with a PMI, whereby the outpatient perinatal mental health unit plays a crucial role in diagnostics and assessment and arranging further treatment and care according to the parents’ needs. We have not identified similar initiatives in the other eight states.

## Discussion

This paper presented an overview of PIMHC services available in Austria as of January 2023. We showed that a broad range of care and treatment services is available, addressing mild to more severe symptoms. However, there are substantial regional disparities, with some regions entirely lacking services. This is especially true for high-level specialised services, such as mother–baby beds or hospital outpatient units. The mapping also shows that there is currently no systematic universal screening in place, but more focus has recently been put on standardised prevention via the newly introduced ‘Psychosoziale Beratung’ as part of the ‘Eltern-Kind-Pass’. The ‘Eltern-Kind-Pass’ may also include a screening tool in the long run once the electronic version of the program will be implemented from 2026 onwards [13]. We found different funding patterns, with most services being fully or largely publicly funded, but the stability of funding varies with more volatile funding for providers funded by regional sources. Many professional groups provide the services; however, a formalised interdisciplinary exchange across organisations only exists in exceptional cases. We did not identify documents outlining care pathways to foster integrated care and quality standards. On the contrary, we found that the programs and therapies have different contents and origins, in which the evidence base is not always clear. The lack of care pathways and institutionalised interdisciplinary exchange is particularly problematic in cases of severe and acute PMI, where admissions need to be organised quickly. Additionally, treatment is organised differently, with individual and often pragmatic solutions found by ambitious individuals (e.g. training psychiatric nurses in infant care to support the mother in a mother–baby unit). Although most services do not explicitly exclude fathers or partners, many are implicitly targeted at mothers as indicated by the name of some offers or the primary target group stated.

As outlined in the women’s health report [18] and international guidelines [12], work in the PIMHC area, especially where it addresses the interaction between mother and child, is not a routine adult mental health activity and requires special therapy programs, trained multidisciplinary teams and specific staffing ratios. The current service landscape, therefore, severely restricts access to specialist care for parents in most Austrian regions. Recommendations based on Irish experiences suggest having one mother–baby hospital unit with six beds per 15,000 deliveries [12]. This would equal around 32 beds within Austria, 22 more than currently available. The restricted capacities require careful prioritisation of admissions and make admissions requiring long-term treatment (e.g. attachment therapies) challenging, as they are blocking the few beds needed for acute cases. Alternative settings (e.g. day-clinic, home treatment) may need to be established or existing therapists may have to be better mobilised and coordinated for therapies, which may not necessarily require inpatient admission. Currently, most units provide one to two beds, and the largest has four beds. On such a small scale, international staffing requirements cannot be fulfilled. The units are also not covered by current DRGs for reimbursement, which means a financial disincentive for providers to admit mothers and babies.

In addition to capacities, access is restricted because of a lack of comprehensive and easily accessible public information on which services or professionals specialise in PIMHC in Austria and due to language restrictions. While the proportion of public funding is high, there are still regional variations regarding private payments (e.g. for midwifery homecare or psychotherapy). For some services (e.g. child and youth welfare), access restrictions are due to referral procedures and entitlement regulations. Even in states with mother–baby beds, PIMHC is limited in situations where the mother’s illness makes her unable to care for the child, while severe PMI is often one of the few indications for inpatient admission to a mother–baby unit in other countries [4].

The current situation contrasts with the use of mental health benefits among mothers in the perinatal period. An analysis of five core mental health benefits funded by the Austrian health insurance (ÖGK) showed that almost one in five mothers (*n* = 23,314) who gave birth between 2017 and 2018 used at least one of those benefits [19]. These figures correspond to international prevalence data, although only a part of available mental health services has been covered in our data and service uptake is usually lower than prevalence [1, 20]. More than 700 mothers were in hospital inpatient or daycare with an ICD-10‑F diagnosis one year after birth across all states, while only ten regular mother–baby beds were available in three states. Furthermore, more than 8,000 women visited a community-based psychiatrist at least once during the perinatal period. Yet, there are very few psychiatrists with special training in PIMHC available, and those are difficult to access because their specialization is not registered.

Regarding professional groups involved, we found that psychologists, psychotherapists, and professionals from the pedagogue spectrum play a crucial role. This contrasts with international recommendations, where midwifery and nursing specialities (e.g. perinatal mental health midwives, maternal and child health nurses, community psychiatric nurses) are supposed to be part of the teams together with medical specialists (e.g. gynaecologists, psychiatrists) [12].

The service landscape reflects gender roles on the societal level, expecting mothers to be the primary carer by putting the mother–infant relationship and less the parent–infant relationship at the focus of support. Parents in nontraditional forms of families are also not actively addressed, e.g. regarding information on whether safe spaces are provided for families where parents are same sex or who have adopted babies.

Our paper has some limitations. Services in PIMHC are heterogeneous across Austria. Although we tried to identify as many available services as possible, some may remain overlooked. We explicitly excluded services offering out-of-home placement, although some may accommodate mothers/parents with PMI with their babies. In some cases, it was difficult to determine whether the offer was for prevention, early detection or treatment because some fall into more than one category (e.g. ‘Frühe Hilfen’). Furthermore, when organisations offer general health and social care programs, we included their services when there was evidence that parents with PMI comprise a proportion of their clients. However, this information was not always available. We have collected the number and types of services and some key characteristics but are aware that these data tell little about the quality of care.

## Conclusions

The results indicate an urgent need to reduce the regional disparities regarding specialised PIMHC, ensuring adequate referrals and treatment and reducing inequalities in access to care. Capacities need to be adapted based on international standards and regional needs, considering quantities and qualities (e.g. skills of staff) of services. Instead of reinventing the wheel in every Austrian region, cross-regional planning and regular interdisciplinary exchange are recommended. The long-standing recommendations on prevention and screening as part of the ‘Eltern-Kind-Pass’ need to be implemented to raise perinatal mental health literacy and improve early identification. Pathways of care need to be defined so that timely support can be organised after a mental health problem is detected.

The results also call for a national strategy and defined political, administrative and service provider responsibilities. Such a strategy needs to address quality standards and workforce qualification/professional development for professional groups having contact with expecting parents and children up to one year of age. The strategy needs to go beyond health, to tackle structural perinatal mental health risk factors such as domestic violence or poverty. This policy prioritization of PIMHC is supported by the prevalence of PMI, compared to other health problems during the perinatal period, the immediate and long-term consequences this has for the children and society, and the current uptake of mental health benefits during the perinatal period.
